# Influence of spray dried porcine plasma in starter diets associated with a conventional vaccination program on wean to finish performance

**DOI:** 10.1186/s40813-016-0021-6

**Published:** 2016-02-08

**Authors:** Joan Pujols, Joaquim Segalés, Javier Polo, Carmen Rodríguez, Joy Campbell, Joe Crenshaw

**Affiliations:** 1 IRTA, Centre de Recerca en Sanitat Animal (CReSA, IRTA-UAB), Campus de la Universitat Autònoma de Barcelona, 08193 Bellaterra, Barcelona, Spain; 2 UAB, Centre de Recerca en Sanitat Animal (CReSA, IRTA-UAB), Campus de la Universitat Autònoma de Barcelona, 08193 Bellaterra, Barcelona, Spain; 3 Departament de Sanitat i Anatomia Animals, Universitat Autònoma de Barcelona (UAB), 08193 Bellaterra, Barcelona, Spain; 4 APC EUROPE, S.A. Avda, Sant Julià 246-258, Pol. Ind. El Congost, E-08403 Granollers, Spain; 5 APC Inc., 2425 SE Oak Tree Court, Ankeny, IA 50021 USA

**Keywords:** Spray dried plasma, *Mycoplasma hyopneumoniae*, Porcine circovirus type 2, Pigs, Vaccination, Weaning stress, Antibody, Carcass

## Abstract

**Background:**

Conventional vaccination programs using a single injection of a combined vaccine against porcine circovirus type 2 (PCV2) and Mycoplasma hyopneumoniae (MHYO) can promote a strong immune response that reduces feed intake for 24 to 48 h post injection. Often such vaccines are given around the time of weaning during a critical stress period in which feed intake is already compromised. Spray dried porcine plasma (SDPP) is a protein source used in starter diets that increases post-weaning feed intake of pigs. The objectives of this study were to determine the effects of a conventional vaccination program along with feeding SDPP in a starter diet on antibody development and wean to finish performance of pigs.

**Results:**

Pigs fed the starter diet with SDPP had improved body weight, average daily weight gain and average daily feed intake during the initial 14 d after weaning along with improved feed efficiency during the initial 7 d after weaning and these responses were independent of vaccination. Vaccination at 3 d after weaning had no significant effect on performance during the initial 14 d after weaning. Cumulative mortality was reduced for pigs fed the starter diet with SDPP, while vaccinated pigs had reduced mortality from d 48 to 145. Both vaccinated pigs and those fed the starter diet with SDPP had heavier carcass weight. One pig per pen was challenged with PCV2 at d 63. A higher percentage of vaccinated pigs were sero-positive for antibodies against PCV2 and MHYO at d 35, 63 and 78. Antibody values against PCV2 were higher for vaccinated pigs at d 35 and 63, but lower at d 146. Percentage of positive samples for PCV2 genome in serum was reduced for vaccinated pigs at d 117 and 146. Antibody values against MHYO were increased for vaccinated pigs at d 35, 63 and 78.

**Conclusions:**

Vaccination supported a long term antibody response against PCV2 and a moderate but weaker antibody response against MHYO for early finishing pigs challenged with PCV2. Using SDPP in the starter diet along with vaccination supported the best long-term beneficial effects on survival to market and carcass weight.

## Background

Spray dried porcine plasma (SDPP) or spray dried bovine plasma (SDBP) has been used in nursery pig diets due to its documented beneficial effects on post-weaning growth, feed intake, morbidity indices and survival [1, 2]. In past studies, under field conditions, pigs suffering from porcine circovirus type 2-systemic disease (PCV2-SD, formerly known as post-weaning multi-systemic wasting syndrome) had improved performance and survival when fed diets containing SDPP [3, 4]. PCV2 is the essential causative agent of PCV2-SD, a multifactorial disease with a severe economic impact worldwide. PCV2-SD is characterized by wasting, decreased weight gain, lymphadenopathy and dyspnea, affecting mainly pigs from 6 weeks of age to market [5].

*Mycoplasma hyopneumoniae* (MHYO) is the main etiological agent of enzootic pneumonia, a chronic respiratory disease that affects mainly growing and finishing pigs. MHYO and PCV2 are potential etiological contributors of the porcine respiratory disease complex, which involves bacterial as well as viral agents [6].

Experimental co-infection with MHYO and PCV2 resulted in a more severe clinical disease in growing [7, 8] and adult [9] animals, although not always [10]. In all cases, PCV2 and MHYO vaccines have demonstrated good results in eliciting antibody responses, reduction of clinical signs and better productive outcome [11–13]. In general, manufacturers recommend the application of these vaccines around the time of weaning.

However, pigs at weaning are subjected to complex changes that can affect pig adaptation and growth. Vaccination against PCV2 and MHYO at weaning may result in a transitory reduced feed intake and growth rate [14–16]. For such a reason, products containing spray dried plasma proteins and digestible energy given in drinking water to weaned pigs have been used to overcome the undesirable effects on growth performance after injection with a PCV2/MHYO vaccine [15]. There is growing evidence in pigs and other species that nutrition before and after weaning can have long-term effects on gut, microbiota interaction and immune development [17]. Therefore, for the present study our objectives were to determine the effects of SDPP in a starter diet fed to pigs injected at 3 d after weaning with a single combined vaccine against PCV2 and MHYO on wean to finish performance, carcass parameters, and detection of pathogen genome and antibody development against PCV2 and MHYO.

## Results

Treatment groups consisted of vaccinated (V) or saline (S) injected pigs on d 3 after weaning, which were fed starter diets with (P) or without (C) SDPP for the initial 14 d post-weaning. Pigs were assigned to 4 treatment groups (1 VC, 2 VP, 3 SC, 4 SP) with a 2 × 2 factorial arrangement of treatments to determine the effects on wean to finish performance and carcass results and serological results over various time periods. Three orthogonal treatment comparisons were used to test for the main effects of vaccination (vaccine vs saline injected pigs; treatment groups 1 VC + 2 VP vs treatment groups 3 SC + 4 SP), starter diet (pigs fed 0 % vs 6 % SDPP in starter diets; treatment groups 1 VC + 3 SC vs treatment groups 2 VP + 4 SP), and the interaction of the main effects of vaccination and starter diet (treatment groups 1 VC + 4 SP vs treatment groups 2 VP + 3 SC).

### Nursery performance results

Average pen performance variables by treatment group and periods of the experiment while pigs were housed at the *Institut de Recerca i Tecnologia Agroalimentàries* (IRTA) nursery facilities are presented in Table 1. The treatment comparison for the main effect of starter diet indicated that average daily weight gain (ADG) was higher for pigs fed starter diet with SDPP during d 0–7 and 0–14 post-weaning. Average daily feed intake (ADFI) was higher for pigs fed starter diet with SDPP during d 0–7, 7–14 and 0–14. Feed efficiency (GF) was improved for pigs fed starter diet with SDPP during d 0–7; but GF was reduced during d 7–14. Treatment comparisons for the main effect of vaccination and the interaction of vaccination and starter diet were not significant for ADG, ADFI or GF during the initial 14 d after weaning or during any other time periods while pigs were housed in nursery facilities. After d 14 when all pigs were fed a common diet to the end of the nursery phase (d 14–48) and for the entire nursery period (d 0–48), there were no significant effects on ADG, ADFI or GF due to treatment comparisons for the main effect of starter diet, vaccination or interaction of starter diet by vaccination.

**Table 1:** Nursery performance of vaccinated pigs fed starter diet with porcine plasma^a^

		Treatment group					Probability *F*-test			
Variable	Day^b^	1 VC	2 VP	3 SC	4 SP	SEM	Trt	Vac	Diet	VxD
ADG, g	0–7	57.6	105.8	70.0	104.4	6.57	<0.01	0.41	<0.01	0.31
ADFI, g	0–7	83.1	111.6	86.9	107.0	4.77	<0.01	0.94	<0.01	0.38
GF	0–7	0.730	0.941	0.805	0.965	0.063	<0.01	0.34	<0.01	0.64
ADG, g	7–14	176.9	180.9	183.4	189.1	8.47	0.77	0.39	0.57	0.92
ADFI, g	7–14	222.2	245.6	223.9	249.3	9.10	0.08	0.76	0.01	0.91
GF	7–14	0.812	0.742	0.811	0.761	0.029	0.22	0.76	0.04	0.73
ADG, g	0–14	117.3	143.3	126.7	146.8	5.28	<0.01	0.23	<0.01	0.58
ADFI, g	0–14	152.7	178.6	155.4	178.2	5.67	<0.01	0.84	<0.01	0.78
GF	0–14	0.763	0.804	0.812	0.827	0.024	0.27	0.14	0.24	0.57
ADG, g	14–48	440.8	448.4	449.8	433.8	8.95	0.57	0.75	0.64	0.20
ADFI, g	14–48	651.9	656.1	650.6	647.0	13.5	0.97	0.70	0.98	0.78
GF	14–48	0.678	0.684	0.694	0.672	0.009	0.39	0.82	0.37	0.14
ADG, g	0–48	346.4	359.4	355.5	350.1	6.83	0.55	0.99	0.58	0.19
ADFI, g	0–48	506.3	516.8	506.2	510.3	10.5	0.87	0.75	0.48	0.76
GF	0–48	0.687	0.696	0.705	0.688	0.009	0.45	0.59	0.67	0.14

^a^Values are least squares mean of the pen average performance variable analyzed for block and treatment group (Trt) using orthogonal treatment comparisons for the effect of vaccination (Vac), starter diet (Diet) and interaction of vaccination and starter diet (VxD). Initial BW (5.83 ± 0.1 kg) was included as a covariant. Starter diets were fed for the initial 14 d after weaning, and then all pigs were fed common diets from d 14 to 48. There were 13 pens per treatment group. Treatment groups were a single injection at d 3 post-weaning with PCV2/MHYO vaccine (V) or saline (S) and starter diet with 0 % (C) or 6 % spray dried porcine plasma (P)

^b^Day or period of experiment

### Mortality and morbidity

Mortality rate of pigs (Table 2) during the initial 14 d after weaning was not significant for any of the treatment comparisons. During d 14–48 and 0–48, mortality rate was reduced for groups of pigs fed starter diet with SDPP. During d 48–145, vaccinated pigs had reduced mortality rate compared to non-vaccinated groups. Over the entire wean to finish period (d 0–145), mortality rate was reduced for groups of pigs fed the starter diet with SDPP. There were no significant interactions of vaccination and starter diet on mortality rate during any periods of the study. Female pigs had lower (*P* <0.01) mortality rate d 14–48 (1.7 % vs 8.3 %), d 0–48 (2.2 % vs 8.3 %) and d 0–145 (3.8 % vs 11.1 %) compared to male pigs (data not shown).

**Table 2:** Mortality, BW and carcass data of vaccinated pigs fed starter diets with porcine plasma^a^

		Treatment group					Probability *F*-test			
Variable	Day^b^	1 VC	2 VP	3 SC	4 SP	SEM	Trt	Vac	Diet	VxD
Pigs, *n*	0	91	91	91	90	--	--	--	--	--
Mortality, %	0–14	1.09	0.00	0.00	0.00	0.55	0.41	0.32	0.32	0.32
	14–48	8.89	4.37	5.53	1.11	2.26	0.11	0.14	0.05	0.98
	0–48	9.95	4.37	5.53	1.11	2.32	0.06	0.10	0.03	0.80
	48–145	1.32	0.00	4.77	3.36	1.66	0.17	0.03	0.40	0.98
	0–145	11.06	4.37	9.95	4.44	2.74	0.17	0.85	0.03	0.82
BW, kg	0	5.84	5.83	5.82	5.84	0.10	0.99	0.96	0.97	0.89
	7	6.24	6.58	6.32	6.57	0.06	<0.01	0.52	<0.01	0.44
	14	7.49	7.84	7.60	7.90	0.09	<0.01	0.36	<0.01	0.73
	21	9.17	9.62	9.53	9.66	0.14	0.06	0.15	0.04	0.27
	35	15.06	15.64	15.39	15.55	0.25	0.38	0.62	0.22	0.57
	48	22.47	23.14	22.90	22.70	0.44	0.63	0.98	0.53	0.24
	63	33.32	34.56	34.90	34.09	0.54	0.46	0.84	0.22	0.29
	83	49.64	50.48	49.46	49.92	0.73	0.76	0.60	0.36	0.79
	114	73.40	75.20	72.22	72.14	1.12	0.16	0.06	0.44	0.39
	145	100.7	102.3	98.67	100.8	1.20	0.18	0.13	0.11	0.83
Carcass, *n* ^c^	159	70	74	72	76	--	--	--	--	--
BW, kg^d^	145	101.7	104.2	98.60	101.8	1.14	<0.01	0.01	0.01	0.77
Carcass, kg	159	86.43	88.70	84.05	86.90	0.94	<0.01	0.03	<0.01	0.75
Lean, %	159	57.97	57.38	58.00	57.48	0.36	0.47	0.85	0.11	0.92

^a^Values are least squares mean of percentage mortality, individual pig body weight (BW) and carcass variable by day or period of experiment analyzed for the effects of sex (S), treatment group (Trt), and interaction of Trt and sex (TxS), using orthogonal treatment comparisons for the main effect of vaccination (Vac), starter diet (Diet) and interaction of vaccination and starter diet (VxD). Initial BW (5.83 ± 0.1 kg) was included as a covariant. Starter diets were fed for the initial 14 d after weaning, and then all pigs were fed common diets from d 14 to 145. Treatment factors were a single injection at d 3 post-weaning with PCV2/MHYO vaccine (V) or saline (S) and starter diet with 0 % (C) or 6 % spray dried porcine plasma (P)

^b^Day or period of experiment

^c^Number of carcasses classified at abattoir differed from number of pigs finished due to culling or lost carcass identification

^d^Live BW of pigs at d 145 that subsequently had carcasses classified on d 159

Although the mortality rate was high, it was similar to that of the source farm and was mainly consistent with bacterial infections. Approximately 70 % of the mortality occurred between d 14 and 48 after pigs were fed a common diet without SDPP, but before challenge with PCV2 inoculum at d 63. Nearly all mortality cases during d 48 to 145 were described as chronic bacterial infections associated with tail-biting or due to euthanasia because of hind limb paralysis.

Necropsies were performed when feasible, but in some cases it was not possible due to the advanced autolysis state of the body. Most deaths were compatible with systemic bacterial infections showing fibrinous polyserositis and arthritis and, less frequently, colisepticemia-like diarrhea and enteritis. A proportion of animals had a chronic course displaying weight loss; those that died showed fibrous polyserositis and pericarditis, craneo-ventral pulmonary consolidation and/or arthritis. Infections by *Haemophilus parasuis*, *Mycoplasma hyorhinis* and *Escherichia coli* could have been involved together with other agents, but specific laboratory investigations were not performed.

The source farm of the herd was sero-positive for PRRSV, and circulation of this virus could potentially have been a predisposing factor for subsequent bacterial infections. However, IDDEX ELISA results for 100 samples taken at d 146 of the study indicated low (12 %) prevalence of samples sero-positive for PRRSV, suggesting PRRSV had not been circulating earlier in the study.

The experimental starter diets and all other diets were non-medicated with the exception of the common transition diet fed from d 48 to 58 when pigs were moved from the nursery to the pre-fattening facility. This diet contained 200 ppm amoxicillin, 24 ppm oxbendazol, and 120 ppm colistin.

Pigs were monitored daily by visual inspection and animal caretakers applied prescribed treatments after veterinarian clinical inspection. Individual pig treatment for polyserositis, arthritis and pneumonia included a product based on Bencylpeniciline procaine 200,000 UI, dihydrostreptomycin (sulfate) 200 mg and dexamethasone (sodium phosphate) 0.5 mg at the recommended dose according to body weight. The percentage of pigs given individual medications (ranged from 9.9 to 16.6 % across treatment groups) was not significantly different for the various treatment group comparisons. At d 26 of the study when several cases of arthritis appeared, Amoxicillin (trihydrate) 70 % at 0.3 g/L was added to drinking water for 5 days.

### Individual pig BW

Results of individual pig BW data by day of experiment from wean to finish (Table 2) indicated that pigs fed the starter diet with SDPP had heavier average BW at d 7, 14 and 21 compared to pigs fed the control diet. However, at all other time periods there were no significant differences due to the main effect of starter diet. Also treatment comparisons for the main effect of vaccination or interaction of starter diet and vaccination were not significant at any time periods of the study. Significant effects for sex (data not shown) indicated female pigs were heavier (*P* <0.03) than male pigs at d 7 (6.51 vs 6.35 kg), d 14 (7.84 vs 7.58 kg) and d 35 (15.67 vs 15.13 kg); but by d 145, males (102.1 kg) were heavier (*P* <0.01) than females (99.1 kg).

### Carcass results

Due to mortality of 27 pigs, lost carcass identification of 36 pigs, and culling or rejection of eight pigs at slaughter, only 292 of the original 363 pigs started on the experiment were used for carcass data (Table 2). Of the eight pigs that were culled at the abattoir, four pigs were culled due to hernias and the other four were culled due to low BW.

There were no significant interactions for the effects of starter diet by vaccination status for any of the carcass parameters. The treatment comparison for the main effect of starter diet indicated that pigs fed the SDPP diet had heavier average carcass weight and average BW at d 145 for pigs that were classified at slaughter compared to the groups fed the control starter diet. Vaccinated pigs had heavier carcass weight and average BW at d 145 for pigs classified at slaughter compared to non-vaccinated pigs. Carcass lean percentage was not significant for the treatment comparisons of the main effects for vaccination or starter diet. Also males had heavier d 145 BW of pigs classified at slaughter (103.1 kg vs 100.1 kg), heavier carcass weight (87.7 kg vs 85.3 kg), and higher carcass lean percentage (58.2 % vs 57.3 %) compared to females (data not shown).

### ELISA values and genome results for PCV2

**Fig. 1:**
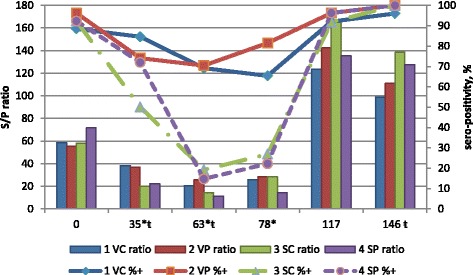
PCV2 ELISA results by treatment and day of experiment. Values are least squares means of treatment group by day of experiment for serum S/P ratio (ratio, bars, left vertical scale) and percentage positive samples (% +, lines, right vertical scale) against PCV2 (26 samples per treatment by day). S/P ratio >17 considered positive. Treatment groups were vaccinated (V) or saline (S) injected pigs on d 3 after weaning that were fed starter diets with (P) or without (C) spray-dried porcine plasma for the initial 14 d post-weaning. One pig per pen was challenged with PCV2 inoculum on d 63 after weaning. Data was analyzed for the effects of sex, treatment group and interaction of treatment group and sex using orthogonal treatment comparisons for the main effects of vaccination (1 VC + 2 VP vs 3 SC + 4 SP), starter diet (1 VC + 3 SC vs 2 VP + 4 SP) and interaction of vaccination and starter diet (1 VC + 4 SP vs 2 VP + 3 SC). ŧ Main effect of vaccination for day 35, 63 and 146 of the experiment for S/P titer (*P* <0.05). *Main effect of vaccination for day 35, 63 and 78 of the experiment for % positive samples (*P* <0.05)

ELISA results for PCV2 S/P ratio and percentage of sero-positive samples by treatment group and day of experiment are presented in Fig. 1. The S/P results >17 were considered positive. The treatment comparisons for the main effect of starter diet or interaction of starter diet and vaccination were not significant at any time period for any of the ELISA results for PCV2. The main effect of vaccination was significant for S/P ratio and percentage sero-positivity for PCV2. Vaccinated pigs had higher percentages of sero-positive samples at d 35, 63 and 78 compared to non-vaccinated pigs. Vaccinated pigs had higher S/P ratio against PCV2 on d 35 and 63, but lower S/P ratio on d 146 compared to non-vaccinated pigs.

**Fig. 2:**
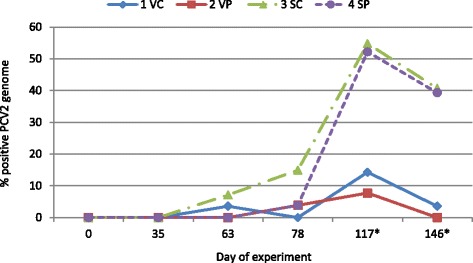
Serum samples positive for PCV2 genome. Values are least squares means of treatment group by day of experiment for percentage of serum samples positive for PCV2 genome (*n* = 26 samples per treatment by day). Treatment groups were vaccinated (V) or saline (S) injected pigs on d 3 after weaning that were fed starter diets with (P) or without (C) spray-dried porcine plasma for the initial 14 d post-weaning. One pig per pen was challenged with PCV2 inoculum on d 63 after weaning. Data was analyzed for the effects of sex, treatment group and interaction of treatment group and sex using orthogonal treatment comparisons for the main effects of vaccination (1 VC + 2 VP vs 3 SC + 4 SP), starter diet (1 VC + 3 SC vs 2 VP + 4 SP) and interaction of vaccination and starter diet (1 VC + 4 SP vs 2 VP + 3 SC). *Main effect of vaccination for day 117 and 146 of the experiment (*P* <0.05)

Percentage of sero-positive samples for PCV2 genome (Fig. 2) was lower for vaccinated pigs at d 117 and 146 compared to non-vaccinated pigs, but no other significant differences were detected at other days of the experiment or for the main effect of starter diet or interaction of starter diet and vaccination.

### ELISA values and genome results for MHYO

**Fig. 3:**
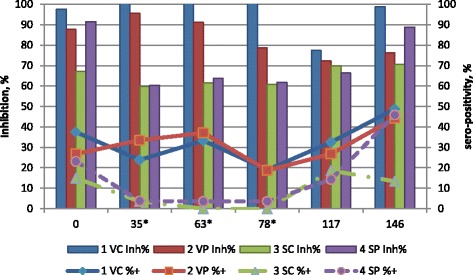
MYHO ELISA results by treatment and day of experiment. Values are least squares means of treatment group by day of experiment for serum inhibition titer (Inh%, bars, left vertical scale) and percentage positive samples (%+, lines, right vertical scale) against MHYO (26 samples per treatment by day). Inhibition percentage >65 was considered positive. Treatment groups were vaccinated (V) or saline (S) injected pigs on d 3 after weaning that were fed starter diets with (P) or without (C) spray-dried porcine plasma for the initial 14 d post-weaning. One pig per pen was challenged with PCV2 inoculum on d 63 after weaning. Data was analyzed for the effects of sex, treatment group and interaction of treatment group and sex using orthogonal treatment comparisons for the main effects of vaccination (1 VC + 2 VP vs 3 SC + 4 SP), starter diet (1 VC + 3 SC vs 2 VP + 4 SP) and interaction of vaccination and starter diet (1 VC + 4 SP vs 2 VP + 3 SC). *Main effect of vaccination for day 35, 63, and 78 of the experiment (*P* <0.05) for both inhibition titer and percentage of positive samples

ELISA results for MHYO inhibition values and percentage of sero-positive samples are presented in Fig. 3. MHYO inhibition results >65 were interpreted as positive. There were no significant effects of starter diet or interaction of starter diet and vaccination on MHYO inhibition values or percentage of sero-positive samples at any time periods. Vaccinated pigs had higher MHYO inhibition value and percentage of sero-positive samples at d 35, 63 and 78 compared to non-vaccinated pigs.

**Fig. 4:**
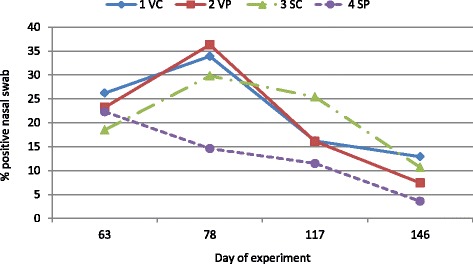
Nasal swab samples positive for MHYO genome. Values are least squares means of treatment group by day of experiment for percentage of nasal swab samples positive for MHYO genome (*n* = 26 samples per treatment by day). Treatment groups were vaccinated (V) or saline (S) injected pigs on d 3 after weaning that were fed starter diets with (P) or without (C) spray-dried porcine plasma for the initial 14 d post-weaning. One pig per pen was challenged with PCV2 inoculum on d 63 after weaning. Data was analyzed for the effects of sex, treatment group and interaction of treatment group and sex using orthogonal treatment comparisons for the main effects of vaccination (1 VC + 2 VP vs 3 SC + 4 SP), starter diet (1 VC + 3 SC vs 2 VP + 4 SP) and interaction of vaccination and starter diet (1 VC + 4 SP vs 2 VP + 3 SC). There were no significant differences detected among treatment groups at any sampling time

There were no significant effects of starter diet, vaccination, or interaction of diet and vaccination on PCR analysis of nasal swabs for MHYO genome at any time period (Fig. 4). Nasal swabs were not sampled on days 0 and 35 of the study.

## Discussion

Weaning of pigs is a period associated with multiple stressors that contribute to reduced growth, feed intake, and feed efficiency and increased morbidity and mortality [18]. Reduced feed intake post-weaning is associated with compromised intestinal barrier function [19]. Weaning stress induces intestinal barrier dysfunctions associated with increased permeability and inflammation that can impact present and future mucosal barrier function [20–22].

Vaccines are useful for helping pigs develop resistance to exposure to pathogens they encounter during their lifetime. However, certain vaccines may stimulate an immune response that results in a measurable reduction of feed intake and growth [12]. When such a vaccine is given around weaning time, this added stress may further exacerbate the reduction in feed intake that is already compromised by weaning stress.

Spray dried plasma has an important role in starter diets as a functional protein source to provide nutrition to support pigs to better transition through the negative effects of weaning stress. The beneficial effects of starter diets containing spray dried plasma on post-weaning pig growth, feed intake, and health are well known [1, 2]. Intestinal barrier dysfunctions associated with weaning stress were reduced when pigs were provided starter diets containing 5 % SDPP fed for 14 d after weaning compared to 0 or 2.5 % SDPP, suggesting diets supplemented with 5 % SDPP support and maintain intestinal barrier function [23]. Therefore, in the present study, 6 % SDPP was used in the starter diet with the intention to assure adequate SDPP was available to support intestinal barrier function during this critical period of post-weaning stress.

In the present study, 6 % SDPP in the starter diet improved short-term post-weaning growth, feed intake and feed efficiency, regardless of vaccination status. Although vaccination at d 3 post-weaning had no significant effect on growth and feed intake, vaccinated pigs fed the control diet had the lowest daily weight gain, feed intake, and gain to feed ratio suggesting a mild short-term effect of the vaccination on growth and feed efficiency during the initial week of the study.

The early growth advantage for pigs fed SDPP during the initial 14 d after weaning was not maintained through the end of the nursery phase (d 48), but by the end of the study both vaccinated pigs and pigs fed starter diet with SDPP had heavier BW and carcass weight. Pigs previously fed SDPP in the starter diet had reduced mortality to d 48 and this reduction of mortality was also maintained to the end of the study. Approximately 70 % of the mortality occurred between d 14 and 48 after pigs were fed a common diet without SDPP, and most of this mortality was associated with lesions compatible with infection caused by *Haemophilus parasuis, Streptococcus suis or Mycoplasma hyorhinis* or other potential pathogens. Vaccinated pigs also had reduced mortality from d 48 to 145. Nearly all mortality cases during d 48 to 145 were described as chronic bacterial infections associated with tail-biting or due to euthanasia because of hind limb paralysis.

Past studies have reported reduced mortality associated with PCV2-SD afflicted nursery and grower pigs fed diets with SDPP [3, 4], and this reduced mortality was extended well beyond the feeding period of diets with SDPP [3]. Also, reduced lung lesions have been reported in pigs fed diets with SDPP and experimentally-infected with swine influenza virus [24]. Furthermore, intestinal permeability of pigs previously fed a diet with 5 % SDPP for 14 d post-weaning was reduced when subsequently challenged with *Salmonella typhimurium* and fed diets without SDPP [25]. Collectively, these past results suggest SDPP in starter diets has a longer-term impact on animal resilience to subsequent stress, and this may be related to the reduction of intestinal barrier dysfunction associated with early life stress.

Infectious pressure for PCV2 (Fig. 2) was <10 % for all treatment groups up to d 63; but for MYHO genome (Fig. 4), it was 18 to 26 % positive across treatment groups at d 63. In order to increase the infectious pressure for PCV2, one pig per pen was inoculated with PCV2 at d 63. Serum PCV2 genome slowly progressed to a higher percentage of positive samples (>50 %) for non-vaccinated pigs by d 117; however, there was little increase in serum PCV2 genome for vaccinated pigs, suggesting vaccination helped reduce subsequent incidence of PCV2 viremia (Fig. 2).

Vaccination supported a strong PCV2 antibody response (Fig. 1) and a modest MHYO antibody response (Fig. 3) and these results are consistent with others [26–30]. A high percentage of pigs at weaning (d 0) had antibodies to PCV2 likely due to the ubiquitous nature of the virus and its widespread sero-prevalence. Under normal conditions pigs acquire antibodies via colostrum that may last up to 10 weeks of age. Therefore, the observed antibody values for PCV2 at weaning are consistent with maternally derived immunity.

Several factors may affect the seroconversion to MHYO under field conditions, such as a variable proportion of infected animals, elongated time to elicit an immune response, or poor correlation of seroconversion with protection that result in no differences in seroconversion between vaccinated and non-vaccinated pigs [31].

Vaccination also reduced the presence of PCV2 genome in serum, after the challenge with PCV2 and this confirms previous research [32, 33]. These serology results may partially explain the reduced mortality of vaccinated pigs during d 48 to 145 of the study when pigs were challenged with PCV2. Starter diet had no significant effects on antibody or genome results. Under the conditions of this study, vaccination and feeding SDPP in the starter diet resulted in pigs with heavier final BW and carcass weight; however most of the reduced mortality was associated with pigs fed the starter diet with SDPP.

## Conclusions

Vaccination supported a long term antibody response against PCV2 and a weaker antibody response against MHYO for early finishing pigs challenged with PCV2. SDPP in the starter diet along with vaccination supported the best long-term beneficial effects on survival to market and final BW and carcass weight. Both early life vaccination and nutrition supplemented with SDPP should be considered for reducing risks of longer term mortality and morbidity in later life stages.

## Methods

### Animals and housing

Pigs ([Large White × Landrace] × Pietrain) weaned at 21 ± 2 d of age were selected from a conventional sow farm managed in a three-week batch farrowing system and transported to an experimental farm at IRTA. The sow farm was seropositive against PRRSV at the time pigs were weaned. Pigs were assigned to 4 treatment groups (1 VC, 2 VP, 3 SC, 4 SP) in a 2 × 2 factorial arrangement of treatments with the effects of injection at d 3 post-weaning with a PCV2/MHYO combined vaccine (V) versus saline (S) or a starter diet with 0 % (C) versus 6 % (P) SDPP as the primary treatment factors. Maternal origin, sex, and weaning weight were used as blocking factors, according to normal procedures at IRTA. Sow parity was not directly used as a blocking factor; however, parity was balanced across treatment groups within block, since it was associated with maternal origin as a blocking factor. After final allotment there were 13 pens with six pens of males, six pens of females, and one pen of mixed sex per treatment group. There were seven pigs per pen, except one pen assigned to treatment four (SP) had only six pigs for a total of 363 pigs started on experiment. Pigs were kept in two rooms at the weaning facility for 48 d and individual pig BW and feed data per pen were recorded at specific intervals (Table 3). Each room had two rows of pens with a central corridor (26 pens per room) with complete slatted floors and pen dividers that allowed for direct pig contact between pens. The experimental pigs were the only pigs located at the weaning facility during the time of the study. At d 48, the seven pigs from the original pens at the weaning facility were moved as an intact group to a single pen located in one of three different rooms in a grower facility at the experimental farm and kept there until d 83. The grower facility also had pen dividers that allowed for direct pig contact between pens, however the experimental pigs were the only pigs kept at the grower facility during the time of the study. After day 83, pigs were moved again and finished at a commercial farm until d 159 post-weaning before being transported to a commercial abattoir. The commercial farm had no other pigs located at the finishing facility during this time; however it was necessary to distribute pigs within treatment group into 10 to 12 pigs per pen with all pens located in the same room. Pens at the commercial finisher had solid pen dividers that did not allow for pig contact between pens. From d 48 to 145 only individual pig body weight data was recorded at specific intervals (Table 3). Carcass weight and lean percentage classification was recorded at the abattoir for 292 of the original 363 pigs started on experiment. Experimental procedures for animals were approved by the *Departament de Medi Ambient i Habitatge de la Generalitat de Catalunya*.

**Table 3:** Schedule for data collection, sampling and other experimental events

Day^a^	Pig age, d	Pig weight	Starter diet	Vaccine^b^	Challenge^c^	Blood sample^d^	Nasal swab^e^
0	21	√	√			√	
3	24		√	√			
7	28	√	√				
14	35	√	√				
21	42	√					
35	56	√				√	
48^f^	69	√					
63	84	√			√	√	√
78	99					√	√
83^g^	104	√					
114	135	√					
117	138					√	√
145	166	√					
146^h^	167					√	√

^a^Day of experiment

^b^Pigs were injected with either vaccine or saline

^c^One pig per pen was inoculated intranasally with PCV2 culture

^d^Blood samples were collected from two pigs per pen for ELISA analysis for antibodies against PCV2 and MHYO and PCR for PCV2

^e^Nasal swab samples were collected from two pigs per pen for PCR for MHYO

^f^All pigs were moved from nursery to grower facility at IRTA on d 48

^g^All pigs were moved from grower facility at IRTA to a commercial finishing farm d 83

^h^Carcass data was recorded d 159 for pigs classified at the abattoir

### Experimental starter diets

Two experimental non-medicated diets were fed during the initial 14 d after weaning and were typical of commercial starter diets for weaned pigs in Spain. SDPP was included at 6 % of the test diet (P) and replaced soy protein concentrate used in the control diet (C) on an equal lysine basis. Both starter diets were formulated to contain 1.45 % total lysine and met or exceeded nutrient requirements recommended by IRTA (Table 4). Common non-medicated commercial diets by phase of production were fed from d 14 to d 159 of the study to all pigs, with the exception that the diet fed from d 48 to 58 of the study contained 200 ppm amoxicillin, 24 ppm oxybendazol and 120 ppm colistin.

**Table 4:** Ingredient and calculated nutrient composition of experimental starter diets

Ingredient, %	Control	Test
Barley	49.34	49.34
Wheat	1.58	0.00
Extruded whole soybeans	15.11	15.12
Soybean meal (47 % CP)	2.48	6.99
Spray dried porcine plasma^a^	0.00	6.00
Soy protein concentrate	9.50	0.00
Dried sweet whey	13.72	13.72
Animal fat	5.24	6.09
Dicalcium phosphate	1.84	2.09
Calcium carbonate	0.00	0.04
Salt	0.28	0.00
Vitamin-trace mineral premix^b^	0.25	0.25
L-lysine	0.36	0.21
DL-methionine	0.19	0.15
L-threonine	0.11	0.02
Total, %	100.0	100.0
Calculated nutrients		
Dry matter, %	89.97	89.55
Crude protein, %	21.08	21.08
Metabolizable energy, kcal/kg	3425	3425
Lactose, %	10.00	10.00
Ether extract, %	9.56	10.48
Ash, %	6.05	6.57
Calcium, %	0.73	0.80
Phosphorus, %	0.76	0.85
Digestible phosphorus, %	0.42	0.55
Sodium, %	0.22	0.34
Chloride, %	0.55	0.53
Lysine, %	1.45	1.45
Methionine, %	0.50	0.44
Methionine + Cysteine, %	0.87	0.89
Threonine, %	0.94	0.94
Tryptophan, %	0.26	0.29

^a^AP820™, APC Europe, Granollers, Spain

^b^Provided the following per kg of diet: vitamin A (E-672) 10000 UI; vitamin D_3_ (E-671) 2000 UI; vitamin E (alpha-tocopherol) 25 mg; vitamin B_1_ 1.5 mg; vitamin B_2_ 3.5 mg; vitamin B_6_ 2.4 mg; vitamin B_12_ 20 μg; vitamin K_3_ 1.5 mg; calcium panthotenate 14 mg; nicotinic acid 20 mg; folic acid 0.5 mg; biotin 50 μg; Fe (E-1) (from FeSO_4_·H_2_O) 120 mg; I (E-2) (from Ca(I_2_O_3_)_2_) 0.75 mg; Co (E-3) (from 2CoCO_3_·3Co(OH)_2_·H_2_O) 0.6 mg; Cu (E-4) (from CuSO_4_·5H_2_O) 150 mg; Mn (E-5) (from MnO) 60 mg; Zn (E-6) (from ZnO) 110 mg; Se (E-8) (from Na_2_SeO_3_) 0.37 mg

### Vaccines

A combined PCV2 and MHYO vaccine was used by mixing two vaccines Ingelvac-Circoflex™ and Ingelvac-Mycoflex™ (Combo-Flex) prepared 1:1 according to the manufacturer’s recommendations (Boehringer Ingelheim Vetmedica GmbH, Ingelheim am Rhein, Germany). The mixed vaccines were given as a single injection (2 ml) by intramuscular route in the neck at d 3 post-weaning. These vaccines were selected as the common products used under commercial conditions in the USA and Europe. Non-vaccinated pigs were injected with 2 ml saline solution at d 3 post-weaning.

### PCV2 inocula

Due to low infectious pressure of PCV2 at d 63 post weaning (12 weeks of age), one pig in each pen was challenged with a field strain (SP-10-7-54-13) of PCV2b virus (titer 10^5.24^TCID_50_/ml) replicated in PK-15 cells free of pestiviruses, *Porcine circovirus type 1*, and *Torque teno sus viruses 1* and *2* [34]. PCV2 was inoculated by intranasal route by applying 1 ml of virus culture on each nostril using an intranasal mucosal atomization device (LMA® MAD300 nasal, Teleflex Medical, NC).

### Sampling and analytical procedures

Blood samples and nasal swabs were collected from two pigs per pen (104 total pigs; 28.7 % of all study pigs) at selected intervals throughout the study (Table 3). The same pigs were sampled throughout the experiment to detect infection and seroconversion. A substitute pig from the same pen was sampled if a previously sampled pig had died. All blood by sampling day were centrifuged at 600 g for 15 min at 4 °C and the serum was analyzed for MHYO and PCV2 antibodies using respectively a monoclonal blocking ELISA Oxoid™ Mycoplasma hyopneumoniae (Oxoid LTD, UK) and an indirect ELISA Ingezim Circo IgG 1.1 PCV.K1 (Ingenasa, Spain), following manufacturers’ instructions. The Oxoid™ ELISA detects antibodies against the 74kD outer external protein of MHYO and is considered more sensitive than and at least as specific as other tests [35]. The Oxoid™ ELISA is less useful to measure antibody levels (it measures percentage of inhibition), but in our study working with conventional pigs that may have unspecific serum reactions for other Mycoplasma species [36], the Oxoid™ ELISA kit was selected. For the Oxoid™ ELISA test an inhibition value >65 was considered positive, while for the PCV2 ELISA an S/P ratio >17 was considered positive.

Nasal swabs were re-suspended in 1000 μL of sterile phosphate-buffered saline (PBS) and vigorously vortexed, and 200 μL of the suspension was used for MHYO DNA extraction. DNA was extracted from nasal and serum swab suspensions using BioSprint 96 DNA Blood Kit (Quiagen Hilden, Germany) based on automated magnetic bead extraction system. To assess for potential contamination during the extraction procedure a negative control was included using PBS as an extraction substrate. Serum samples were analyzed for the presence of PCV2 genome by means of a real time quantitative PCR (qrt-PCR) as previously described [37]. Viral concentrations were expressed as PCV2 DNA copy numbers per ml of serum, as described previously [38]. Nested PCR (nPCR) for MHYO were tested by means of a previously described technique [31, 39].

### Statistical analysis

Pen was used as the experimental unit when analyzing the effect of weight block and treatment group. Orthogonal treatment comparisons for the main effects of vaccination (treatment groups 1 VC + 2 VP vs treatment groups 3 SC + 4 SP), starter diet (2 VP + 4 SP vs 1 VC + 3 SC), and interaction of vaccination and starter diet (1 VC + 4 SP vs 2 VP + 3 SC), along with the covariance of initial BW were included in the analysis of variance model (SAS 9.2, SAS Inst. Inc., Cary, NC). Individual pig BW and mortality rate at various intervals from wean to finish and individual pig carcass data were analyzed for treatment group, sex, and interaction of treatment and sex, along with the covariance of initial BW and the orthogonal treatment comparisons previously described. Serology and nasal swab data were analyzed the same as individual pig body weight and carcass data. Results were considered significant at *P* <0.05.
